# The Role of Kidney Biopsy as a Tool for Personalized Treatment Decision-Making in Patients with Anti-Neutrophil Cytoplasmic Antibody (ANCA)-Associated Nephritis

**DOI:** 10.3390/jpm16030153

**Published:** 2026-03-07

**Authors:** Makoto Harada, Shotaro Aso, Takayuki Nimura, Kosuke Yamaka, Daiki Aomura, Aiko Yamada, Kosuke Sonoda, Akinori Yamaguchi, Yutaka Kamimura, Tohru Ichikawa, Mamoru Kobayashi, Koji Hashimoto, Yuji Kamijo

**Affiliations:** 1 Department of Nephrology, Shinshu University Hospital, 3-1-1, Asahi, Matsumoto 390-8621, Japan; 2 Department of Health Services Research, Graduate School of Medicine, The University of Tokyo, 7-3-1, Hongo, Bunkyo-ku, Tokyo 113-8655, Japan; 3 Department of Nephrology, Nagano Red Cross Hospital, 5-22-1, Wakasato, Nagano 380-8582, Japan

**Keywords:** anti-neutrophil cytoplasmic antibody (ANCA), anti-neutrophil cytoplasmic antibody-associated vasculitis (AAV), kidney biopsy, end-stage kidney disease, infectious complications, personalized management strategies

## Abstract

**Background/Objectives**: Personalized treatment approaches are increasingly recognized as essential in the management of anti-neutrophil cytoplasmic antibody-associated vasculitis (AAV), given the substantial heterogeneity in disease severity and patient characteristics. Kidney biopsy has the potential to serve as an effective tool for personalized treatment decision-making in patients with AAV. This study aimed to investigate the association of kidney biopsy with intensive immunosuppressive therapy and clinical outcomes in patients with AAV and kidney impairment. **Methods**: In this retrospective study, propensity score overlap weighting was applied to compare intensive immunosuppressive therapy and clinical outcomes (ESKD, death, combined ESKD and death, and infectious complications) between patients with AAV who underwent kidney biopsy and those who did not. **Results**: Out of 74 patients with AAV, 38 underwent kidney biopsy. Overlap weight analysis revealed that kidney biopsy was significantly associated with intensive immunosuppressive therapy (risk difference [RD], 28.9%; 95% confidence interval [CI], 0.017 to 0.562). Kidney biopsy was not associated with combined ESKD and death (RD, −0.2%; 95% CI, −0.302 to 0.298), death (RD, −3.8%; 95% CI, −0.264 to 0.189), ESKD (RD, −7.3%; 95% CI, −0.353 to 0.207), and infectious complications (RD, −25.9%; 95% CI, −0.537 to 0.020). **Conclusions**: In this observational cohort, kidney biopsy was associated with intensification of immunosuppressive therapy. However, after adjustment using overlap weighting, no statistically significant difference in clinical outcomes was detected, and the reduced effective sample size limited statistical power. These findings should be interpreted cautiously, as causal inference regarding the prognostic impact of kidney biopsy remains limited.

## 1. Introduction

Anti-neutrophil cytoplasmic antibody (ANCA)-associated vasculitis (AAV) is a life-threatening systemic small vessel vasculitis that often causes severe kidney injury due to necrotizing crescentic glomerulonephritis, leading to end-stage kidney disease (ESKD) [1]. In addition to glucocorticoids, the induction of intensive immunosuppressive therapy has recently been reported to contribute to better kidney outcomes in patients with AAV [2].

Kidney biopsy is crucial for diagnosing crescentic glomerulonephritis caused by AAV, assessing its severity, and predicting kidney prognosis in patients with AAV [3,4]. Consequently, kidney biopsy may offer improved prognosis and kidney function through appropriate immunosuppressive therapy in these individuals. However, whether kidney biopsy meaningfully influences treatment decisions and improves clinical outcomes beyond diagnostic confirmation remains unclear [5,6,7]. Nevertheless, kidney biopsy is invasive and carries the risk of not only bleeding from a puncture but also thrombosis accompanied by complete bed rest after the puncture [3,8]. Therefore, kidney biopsy is difficult to perform in some compromised patients, such as the elderly and those taking anticoagulants and/or antiplatelet agents. Furthermore, a substantial proportion of patients with AAV may be older [9,10]. The average age of patients with microscopic polyangiitis (MPA) reportedly ranges from 55 to 75 years, and several previous studies from Japan have reported a mean age of >70 years among patients with AAV [9,10]. Our prior research revealed that only 34% of older patients with AAV underwent kidney biopsy [11] and that the proportion of kidney biopsies performed in older patients with AAV was lower than that in younger patients with AAV [11]. Despite the potential benefits of kidney biopsy in managing AAV-related kidney injury, a significant number of patients face challenges in undergoing this procedure owing to age, medication use, and associated complications. Therefore, it is imperative to understand the utility of kidney biopsy on the treatment decisions and clinical outcomes of patients with AAV.

In the era of personalized medicine, treatment decisions for AAV should be tailored to the individual patient, incorporating serological, clinical, and histopathological data from kidney biopsy. The Berden classification demonstrates how biopsy-derived findings can stratify patients by prognosis. However, when a kidney biopsy is not feasible, as is often the case with older patients or those at high risk for bleeding, clinicians must rely on alternative approaches to individualize treatment. Therefore, understanding the real-world impact of kidney biopsy on treatment decisions and outcomes is a critical step toward developing personalized management strategies for patients with AAV. The present study aimed to assess the association between kidney biopsy and treatment intensity, as well as the association between kidney biopsy and clinical outcomes, in patients with AAV. By examining these associations, we sought to inform personalized strategies for the evaluation and treatment of patients with AAV.

## 2. Materials and Methods

### 2.1. Study Design and Patient Selection

This two-center retrospective observational study was conducted on patients who were newly diagnosed with and treated for AAV with kidney impairment at the Department of Nephrology in Shinshu University Hospital from January 2013 to December 2019 and at the Department of Nephrology in Nagano Red Cross Hospital from January 2010 to December 2017. Clinical data were collected from patients’ medical records. AAV was defined according to the algorithm suggested by Watts et al. [12]. Patients were categorized as having granulomatosis with polyangiitis (GPA), MPA, or unclassifiable vasculitis. AAV severity was evaluated using the Birmingham Vasculitis Activity Score version 3 (BVAS-3) upon hospital admission [13]. AAV treatment was provided according to the Japanese guidelines for ANCA-positive rapidly progressive glomerulonephritis [10]. Patients with eosinophilic granulomatosis with polyangiitis (EGPA) were excluded because of the distinct kidney prognosis, treatment patterns, and incidence of kidney impairment in patients with EGPA compared to those with MPA and GPA [1]. Patients with drug-induced AAV or co-existing anti-glomerular basement membrane (GBM) antibody positivity, those aged < 20 years, and those who had received immunosuppressive therapy prior to AAV induction therapy were also excluded.

Patients were divided into two groups: (i) the kidney biopsy group, which comprised patients who underwent kidney biopsy, and (ii) the control group, which included those who did not undergo kidney biopsy. Kidney biopsy was performed within one month after hospital admission to evaluate the cause of kidney impairment and the severity of the kidney lesion, following the procedure described by Sonoda et al. [14]. A nephrologist performed percutaneous kidney biopsy using an automatic biopsy needle under ultrasound guidance. Furthermore, all participants were ANCA-positive at baseline (MPO-ANCA and/or PR3-ANCA), and all patients were clinically suspected of having AAV prior to kidney biopsy. Importantly, kidney biopsy did not reveal alternative or additional diagnoses in this cohort, indicating that biopsy was primarily used for assessment of kidney involvement and severity rather than for diagnostic reclassification.

Data were extracted from a previously published clinical database [11]. This study was conducted in accordance with the principles embodied in the Declaration of Helsinki and was approved by the Institutional Review Board/Ethics Committee of Shinshu University School of Medicine (approval number: 5211, approval date: 28 June 2021). The requirement for written informed consent was waived owing to the retrospective nature of the study.

### 2.2. Outcomes and Baseline Characteristics

The primary outcome was the need for intensive immunosuppressive therapy. The secondary outcomes were the composite of death and ESKD, death alone, ESKD alone, and infectious complications within six months of starting immunosuppressive treatment. Intensive immunosuppressive therapy was defined as the use of immunosuppressive therapy in addition to oral prednisolone. Immunosuppressive therapy included methylprednisolone pulse therapy, cyclophosphamide, and rituximab. Methylprednisolone pulse therapy involved intravenous administration of methylprednisolone (500–1000 mg/day) for three consecutive days. Cyclophosphamide and rituximab were also intravenously administered. ESKD was defined as the need for renal replacement therapy, which included maintenance dialysis for >30 days or kidney transplantation. Infectious complications were defined as infections that required hospitalization and antimicrobial therapy, and were identified based on physician diagnosis through detailed chart review. Microbiological confirmation was used when available but was not mandatory for case definition.

Baseline characteristics included age, sex, body mass index (BMI), diabetes mellitus, interstitial lung lesions, clinical classification of vasculitis, BVAS-3, serum albumin level, estimated glomerular filtration rate (eGFR), C-reactive protein (CRP) level, hemoglobin level, hematuria, proteinuria, and trimethoprim–sulfamethoxazole (TMP-SMZ) use. Diabetes mellitus was defined as a glycated hemoglobin A1c level >6.5%, prescription of insulin or hypoglycemic agents, and/or a documented history of diabetes mellitus. Patients with type 1 or drug-induced diabetes were excluded. Interstitial lung lesions were defined as bilateral interstitial lung lesions on computed tomography images. The maximum prednisolone dose for treatment was adjusted based on the ideal body weight. Rapid prednisolone dose reduction was defined as a decrease in the daily prednisolone dose to <20 mg/day within 8 weeks, as per a previous nationwide Japanese study [3]. Blood and urine samples collected upon hospital admission were also evaluated. Proteinuria was defined as urinary protein level > 0.15 g/gCr. Hematuria was defined as red blood cell sediment count > 5/high-power field. eGFR was calculated using previously reported formulas: eGFR (mL/min/1.73 m^2^) = 194 × serum creatinine (−1.094) × age (−0.287) × 0.739 (if female) [15]. Kidney impairment was defined as eGFR < 60 mL/min/1.73 m^2^ and/or the presence of proteinuria and/or hematuria.

### 2.3. Statistical Analyses

Normally distributed continuous variables are presented as means with standard deviations, whereas categorical variables are expressed as numbers and percentages. Continuous variables were compared between the two groups using the Student’s t-test, whereas categorical variables were compared using Fisher’s exact probability test. Overlap weighting based on propensity scores was applied to adjust for the baseline covariates. Propensity scores for kidney biopsy were calculated using logistic regression based on age, sex, BMI, serum albumin level, BVAS-3, CRP level, eGFR, hematuria, proteinuria, hemoglobin level, presence of diabetes mellitus, interstitial lung lesions, categories of AAV (MPA, GPA, or unclassifiable vasculitis), and TMP-SMZ use. Subsequently, overlap weighting was employed to achieve balance between the groups, as described by Li et al. [16]. The weight for patients who underwent kidney biopsy was 1–propensity score, whereas the weight for patients who did not undergo kidney biopsy was the propensity score. This method emphasizes individuals with a substantial probability of being in either treatment group, thereby targeting the population in which clinical equipoise exists. Consequently, overlap weighting estimates the average treatment effect in a hypothetical subpopulation whose characteristics are a combination of those of the two groups rather than specifically aligning with either group. A standardized mean difference (SMD) of <0.1 was considered well-balanced. Statistical analyses were performed using R (version 4.3.1; Vienna, Austria).

## 3. Results

**Figure 1:**
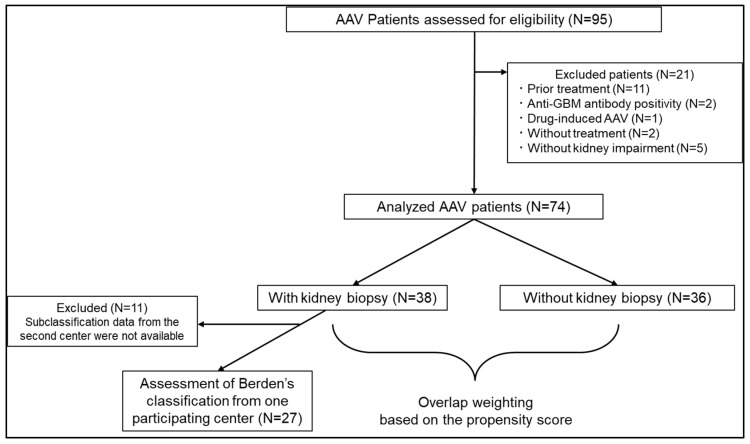
Study flow. Of the 95 eligible patients with AAV, 21 were excluded based on the criteria. Ultimately, 74 patients were analyzed and divided into two groups according to whether they underwent kidney biopsy or not. Subsequently, overlap weighting based on propensity scores was performed. AAV, Anti-neutrophil cytoplasmic antibody-associated vasculitis; anti-GBM, anti–glomerular basement membrane.

Of the 95 eligible patients, 21 were excluded: 11 due to prior treatment, two due to anti–glomerular basement membrane antibody positivity, one due to drug-induced AAV, two due to lack of treatment, and five due to the absence of kidney impairment (Figure 1). The final analysis included 74 patients; of these, 38 patients underwent kidney biopsy, whereas 36 did not (Figure 1). Of 38 patients who underwent kidney biopsy, pathological subclassification was available for 27 patients from one participating center (Figure 1). For the remaining 11 patients from the second center, subclassification data were not available in a retrievable format at the time of analysis due to institutional data access restrictions.

Prior to overlap weighing, background characteristics such as age, sex, BMI, interstitial lung lesions, clinical classification of vasculitis, BVAS-3, laboratory data, and TMP-SMX use were not balanced between patients who underwent kidney biopsy and those who did not (Table 1).

**Figure 2:**
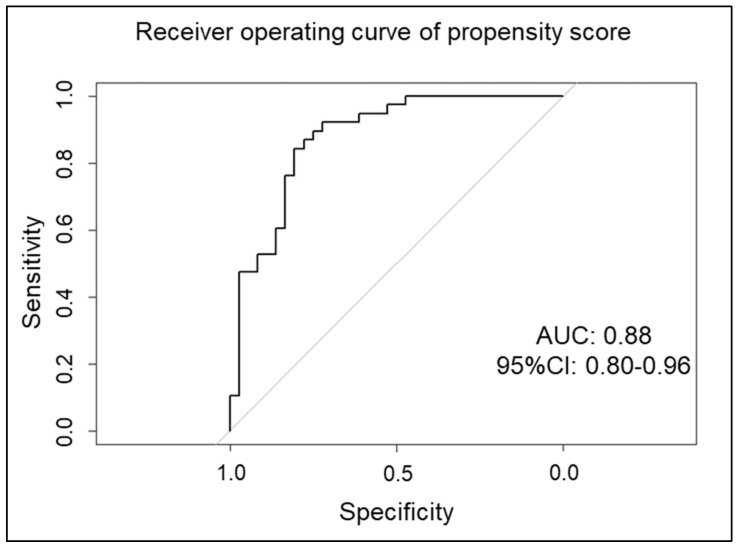
Receiver operating curve of propensity scores. This curve was drawn using age, BMI, sex, serum albumin level, BVAS-3, C-reactive protein level, eGFR, hematuria, proteinuria, hemoglobin level, presence of diabetes, presence of interstitial lung lesions, categories of AAV (MPA, GPA, or unclassifiable vasculitis), and TMP-SMZ use as variables. BMI, body mass index; BVAS-3, Birmingham Vasculitis Activity Score version 3; eGFR, estimated glomerular filtration rate; AAV, Anti-neutrophil cytoplasmic antibody-associated vasculitis; MPA, microscopic polyangiitis; GPA, granulomatosis with polyangiitis; TMP-SMZ, trimethoprim–sulfamethoxazole.

**Figure 3:**
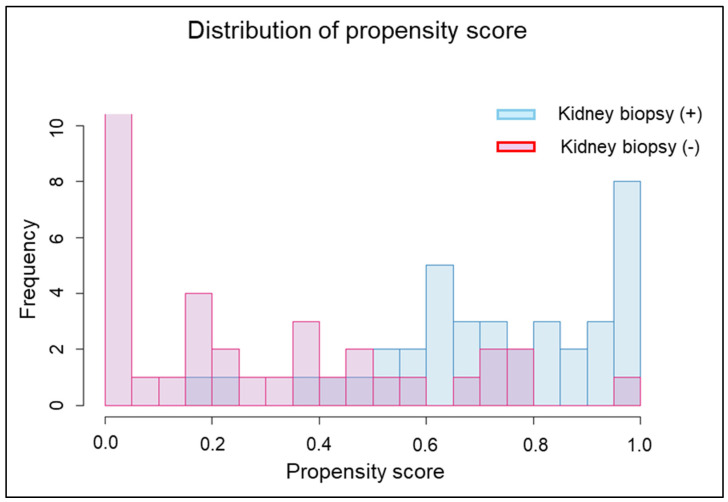
Distribution of propensity scores. Some patients in both groups had overlapping propensity scores.

Overlap weight analysis based on propensity scores indicated an area under the curve of 0.88 (95% confidence interval [CI], 0.80–0.96) for this model (Figure 2). The distribution of propensity scores is presented in Figure 3. After overlap weighting, all covariates were well-balanced between patients who underwent kidney biopsy and those who did not (Table 1).

Kidney biopsy was associated with an increase in intensive immunosuppressive therapy (risk difference [RD], 28.9%; 95% CI, 0.017 to 0.562; *p* = 0.038). Comparisons of treatment details showed that the overall use of intensive immunosuppressive therapy was higher in patients who underwent kidney biopsy. After overlap weighting, SMDs for individual treatment components varied. Intravenous methylprednisolone and rituximab were more frequently used in the biopsy group and showed relatively large SMDs, indicating residual imbalance. In contrast, cyclophosphamide demonstrated small SMDs, suggesting good balance between groups. Plasma exchange also showed relatively large SMD, but was less frequently used in the biopsy group. Overall, no uniform pattern was observed across specific therapies (Supplementary Table S1). Kidney biopsy was not associated with a reduction in the composite of death and ESKD (RD, −0.2%; 95% CI, −0.302 to 0.298; *p* = 0.99), death alone (RD, −3.8%; 95% CI, −0.264 to 0.189; *p* = 0.74), ESKD alone (RD, −7.3%; 95% CI, −0.353 to 0.207; *p* = 0.60), and infectious complications (RD, −25.9%; 95% CI, −0.537 to 0.020; *p* = 0.07) (Table 2). Although the difference did not reach statistical significance, the risk difference of −25.9% may suggest a trend toward a higher incidence of infectious complications. These results were similar to those obtained before overlap weighting based on propensity scores (Supplementary Table S2).

The most common reasons for the non-performance of kidney biopsy were older age, reduced cognitive abilities, and reduced activities of daily living following the use of anticoagulants and/or antiplatelet agents (Table 3). One patient required red blood cell transfusion due to the progression of anemia; however, no other complications associated with the kidney biopsy were observed.

## 4. Discussion

The present study demonstrated that patients with AAV who underwent kidney biopsy were more likely to receive intensive immunosuppressive therapy. However, after adjusting for statistical differences using overlap propensity score weighting, no significant differences in short-term clinical outcomes were detected between patients who underwent biopsies and those who did not. Importantly, the markedly reduced effective sample size after weighting indicates limited statistical power; therefore, the findings should be interpreted cautiously.

Although previous studies and current guidelines have shown that combination therapy with glucocorticoids and additional immunosuppressive agents improves outcomes compared with glucocorticoid monotherapy, our cohort consisted predominantly of older adults (mean age 75 years) [5,6,17]. In this clinical context, treatment intensification may confer both potential benefits and increased risks. While the observed difference in infectious complications did not reach statistical significance, the direction and magnitude of the risk difference (the risk difference of −25.9%) warrant cautious interpretation, particularly in frail older populations in whom vulnerability to treatment-related adverse events may be heightened.

Importantly, the absence of a statistically significant short-term benefit in this study should not be interpreted as evidence against the clinical usefulness of kidney biopsy. Rather, the findings reflect the complexity of real-world treatment decision-making processes and the limitations of observational comparisons between groups of patients with different clinical characteristics.

Regarding glucocorticoid therapy, all patients received a standard-dose regimen, and no patient received a reduced-dose protocol during the study period [18]. Prednisolone dosing adjusted for ideal body weight was similar in the biopsy and non-biopsy groups. Given the emerging evidence supporting reduced-dose glucocorticoid strategies, the consistent use of standard-dose regimens in both groups may have obscured potential differences in outcomes related to biopsy-related treatment decisions [18].

According to the 2016 EULAR/ERA-EDTA recommendations, kidney biopsy is advised for diagnostic confirmation and prognostic assessment in patients with AAV; however, the level of evidence is graded as 3 and the strength of recommendation as C [5,6]. Moreover, there is currently insufficient evidence demonstrating that pathological findings in AAV-associated nephritis directly influence the choice or intensity of immunosuppressive therapy [5,6,7]. Thus, how histopathological information should be incorporated into therapeutic strategies remains an unresolved issue.

To explore this issue descriptively, we performed an exploratory analysis of treatment patterns and outcomes according to Berden’s histopathological classification among patients who underwent kidney biopsy [4]. This analysis was restricted to 27 patients for whom detailed subclassification data were available, and several pathological categories were represented by very small numbers. Within this limited sample, treatment intensity and prednisolone dosing appeared broadly similar across pathological classes, and outcome differences were not consistent. Given the small subgroup sizes and limited statistical power, these observations cannot support definitive conclusions regarding pathology-guided treatment stratification and should be regarded as hypothesis-generating for future studies.

A key consideration in interpreting these findings is the short-term nature of the primary endpoints. Outcomes assessed within six months primarily captured early mortality and infectious complications and may be insufficient to fully evaluate long-term renal survival in AAV, where relapse and slow progression of chronic kidney disease are key determinants of prognosis. Potential benefits of biopsy-guided treatment tailoring, such as avoidance of overtreatment in patients with irreversible lesions or prevention of late CKD progression in those with active but potentially reversible disease, may not become apparent until 12–24 months or longer. Therefore, the absence of short-term benefit observed in this study does not exclude the possibility of longer-term advantages associated with biopsy-guided management.

Furthermore, this study was conducted before the widespread adoption of reduced-dose glucocorticoid regimens and complement C5a inhibitors, such as avacopan [19]. With the introduction of these therapies, which allow for substantial reduction in glucocorticoid exposure, the therapeutic risk–benefit balance in frail older patients with AAV may shift [19]. In such settings, kidney biopsy may assume an increasingly important role by providing diagnostic and prognostic certainty to support the appropriate use of high-cost, low-toxicity targeted therapies, while avoiding unnecessary immunosuppression in patients unlikely to benefit. In this evolving therapeutic landscape, biopsy-guided risk stratification has been proposed as a cornerstone of personalized medicine for AAV. This approach may allow clinicians to adjust treatment intensity based on each patient’s unique disease characteristics.

From a personalized medicine perspective, kidney biopsy may be most valuable in patients with diagnostic uncertainty, potentially reversible renal lesions, or younger patients in whom long-term renal survival is a major concern. In contrast, frail older patients with multiple factors limiting the feasibility of kidney biopsy, as summarized in Table 3, may be less likely to derive meaningful benefit from invasive diagnostic procedures. The decision of whether to perform kidney biopsy should be individualized based on each patient’s clinical profile, procedural risk, and expected therapeutic impact. In the present study, clinicians may have appropriately identified such patients as unlikely to benefit from biopsy or aggressive treatment and selectively refrained from performing kidney biopsy. From this viewpoint, the avoidance of biopsy in these cases may represent a favorable aspect of individualized, real-world clinical decision-making in AAV care.

Some limitations of this study should be acknowledged. First, this was a retrospective observational study conducted at a single center with a relatively small sample size, limiting generalizability and precluding definitive causal inference. Although overlap propensity score weighting was used to adjust for baseline differences, residual or unmeasured confounding cannot be excluded.

Variables included in the propensity score model were selected based on clinical relevance and their potential influence on both the likelihood of undergoing kidney biopsy and subsequent treatment decisions. Major organ involvement was considered; however, certain indicators of disease severity could not be included. Pulmonary hemorrhage was not adjusted for because of its very low incidence in this cohort. Data on oxygen requirements at admission were unavailable. Although a small number of patients required ICU admission at treatment initiation, ICU admission criteria were not standardized, and some patients who might otherwise have been managed in the ICU were treated in general wards. Given these limitations and the small number of events, ICU admission was not included as a covariate. Consequently, unmeasured or residual confounding related to disease severity may have influenced the results.

Second, treatment decisions were not standardized and were left to the discretion of attending physicians. Variations in clinical judgment, institutional practice patterns, and patient preferences may therefore have influenced both the decision to perform kidney biopsy and subsequent treatment intensity.

Third, an additional limitation relates to the availability of detailed histopathological subclassification data. Although 38 patients underwent kidney biopsy, Berden classification was available for 27 patients from one participating center. Subclassification data from the second center were not available in a retrievable format at the time of analysis due to institutional data management procedures. Consequently, pathology-based subgroup analyses were restricted to a subset of biopsied patients, which may introduce selection bias and limit the generalizability of these exploratory findings. Exploratory analyses of pathological subgroups were limited by small sample sizes and should not be interpreted as evidence of inadequate treatment stratification. These observations are now presented as generating hypotheses and may serve as a basis for future research.

Fourth, the observation period focused on early outcomes within six months and may not have captured long-term renal survival, relapse, or late adverse events. Future multicenter prospective studies with larger sample sizes, longer follow-up, and incorporation of contemporary steroid-sparing therapies are needed to clarify how kidney biopsy findings should be optimally integrated into personalized management strategies for patients with AAV.

Fifth, this study is subject to confounding by indication, which is inherent to its observational design. Patients who did not undergo a kidney biopsy were generally older and frailer and had more medical contraindications. This reflects real-world clinical decision-making rather than random allocation. Despite adjustment using overlap weighting, the high area under the curve (AUC 0.88) and the significantly smaller effective sample size suggest significant clinical differences between the two groups. Therefore, comparing “fit-for-biopsy” and “unfit-for-biopsy” patients fundamentally limits causal inference and generalizability, as well as constraining the ability to evaluate the intrinsic prognostic utility of kidney biopsy itself.

## 5. Conclusions

In the present study, the performance of kidney biopsy was associated with intensification of immunosuppressive therapy. However, no statistically significant difference in short-term clinical outcomes was detected after overlap weighting, and the markedly reduced effective sample size limited statistical power. The high AUC and the fundamental differences in baseline characteristics between the two groups indicate that biopsy and non-biopsy patients represent two clinically distinct populations, substantially limiting causal inference. From a personalized medicine perspective, the contribution of this study lies in characterizing real-world patient profiles in which kidney biopsy is or is not performed, rather than demonstrating a uniform benefit of biopsy itself. These findings suggest that the decision to perform kidney biopsy should be individualized: biopsy-derived histopathological information may guide more targeted treatment in suitable candidates, whereas forgoing biopsy may represent an appropriate clinical judgment in frail older patients with multiple contraindications. These findings should be interpreted with caution and regarded as hypothesis-generating. Future prospective studies with larger sample sizes and longer follow-up are needed to establish personalized decision-making frameworks for kidney biopsy in patients with AAV.

## Figures and Tables

**Table 1:** Baseline characteristics of the study participants.

	Before Overlap Weighting					After Overlap Weighting				
	Kidney Biopsy (−)		Kidney Biopsy (+)		SMD	Kidney Biopsy (−)		Kidney Biopsy (+)		SMD
	*N* = 36		*N* = 38			*N* = 10.3		*N* = 10.3		
Age, years (SD)	78	(9)	72	(9)	0.67	75	(9.7)	75	(7.2)	<0.001
Male, *n* (%)	16	(44.4)	22	(57.9)	0.27	5.7	(55.3)	5.7	(55.3)	<0.001
BMI, kg/m^2^ (SD)	22.0	(3.0)	22.6	(3.4)	0.17	21.8	(2.7)	21.8	(2.7)	<0.001
Diabetes mellitus, *n* (%)	9	(25.0)	9	(23.7)	0.03	2.6	(27.0)	2.6	(27.0)	<0.001
Interstitial lung lesions, *n* (%)	17	(47.2)	20	(52.6)	0.11	5.6	(53.9)	5.6	(53.9)	<0.001
Clinical classification										
GPA, *n* (%)	2	(5.6)	0	(0.0)	0.34	0	(0.0)	0	(0.0)	<0.001
MPA, *n* (%)	30	(83.3)	37	(97.4)	0.49	10.1	(97.5)	10.1	(97.5)	<0.001
Unclassifiable vasculitis, *n* (%)	4	(11.1)	1	(2.6)	0.34	0.3	(2.5)	0.3	(2.5)	<0.001
BVAS-3, score (SD)	15	(5)	16	(4)	0.16	16	(5)	16	(3)	<0.001
Laboratory data										
Albumin, g/dL (SD)	2.5	(0.7)	3.0	(0.5)	0.71	2.8	(0.6)	2.8	(0.6)	<0.001
eGFR, mL/min/1.73 m^2^, (SD)	29.0	(28.7)	23.3	(17.8)	0.24	19.6	(15.5)	19.5	(13.9)	<0.001
CRP, mg/dL (SD)	9.0	(6.8)	4.8	(5.9)	0.66	6.9	(6.3)	6.9	(7.1)	<0.001
Hemoglobin, g/dL (SD)	9.1	(1.9)	10.0	(1.8)	0.45	9.3	(1.9)	9.3	(1.7)	<0.001
Hematuria, *n* (%)	31	(86.1)	36	(94.7)	0.30	9.7	(94.0)	9.7	(94.0)	<0.001
Proteinuria, *n* (%)	30	(83.3)	38	(100.0)	0.63	10.3	(100)	10.3	(100)	<0.001
Treatment pattern										
TMP-SMX use, *n* (%)	30	(83.3)	37	(97.4)	0.49	9.9	(96.4)	9.9	(96.4)	<0.001

The non-integer sample sizes after overlap weighting represent weighted pseudo-samples rather than actual patient counts. BMI, body mass index; BVAS-3, Birmingham Vasculitis Activity Score version 3; CRP, C-reactive protein; eGFR, estimated glomerular filtration rate; GPA, granulomatosis with polyangiitis; MPA, microscopic polyangiitis; SD, standard deviation; SMD, standardized mean difference; TMP-SMX, trimethoprim–sulfamethoxazole.

**Table 2:** Association between kidney biopsy and clinical outcomes within six months and intensive immunosuppressive therapy.

	Risk Difference	95% CI	*p*
Intensive immunosuppressive therapy	28.9%	0.017 to 0.562	0.038
Death or ESKD within six months	−0.2%	−0.302 to 0.298	0.99
Death within six months	−3.8%	−0.264 to 0.189	0.74
ESKD within six months	−7.3%	−0.353 to 0.207	0.60
Infectious complications within six months	−25.9%	−0.537 to 0.020	0.07

CI, confidence interval; ESKD, end-stage kidney disease.

**Table 3:** Reasons why a kidney biopsy was not performed.

	Cases
Age, reduced cognitive abilities, reduced activities in daily living	18
Use of anticoagulants and/or antiplatelet agents	9
Respiratory failure	5
Mild kidney dysfunction and/or scarcity of urinary abnormalities	5
Refusal to undergo kidney biopsy	3
Thrombocytopenia	3
Anemia	3
Deep vein thrombosis	2
Unilateral kidney	1

Each case does not always have only one reason.

## Data Availability

The data used in this study are available from the corresponding author upon reasonable requests.

## Supplementary Materials

The following supporting information can be downloaded at https://www.mdpi.com/article/10.3390/jpm16030153/s1. Table S1: Details of the treatment. The non-integer sample sizes after overlap weighting represent weighted pseudo-samples rather than actual patient counts. ESKD, end-stage kidney disease; mPSL, methylprednisolone; PSL, prednisolone; SD, standard deviation; SMD, standardized mean difference.; Table S2: Association between kidney biopsy and clinical outcomes within six months and intensive immunosuppressive therapy before overlapped weighting based on the propensity score. CI, confidence interval; ESKD, end-stage kidney disease.

## Abbreviations

The following abbreviations are used in this manuscript:

AAV	Anti-neutrophil cytoplasmic antibody-associated vasculitis
ANCA	Anti-neutrophil cytoplasmic antibody
BMI	Body mass index
BVAS	Birmingham Vasculitis Activity Score-3
CI	Confidence interval
CRP	C-reactive protein
CT	Computed tomography
CY	Cyclophosphamide
eGFR	Estimated glomerular filtration rate
EGPA	Eosinophilic granulomatosis with polyangiitis
ESKD	End-stage kidney disease
GBM	Glomerular basement membrane
GPA	Granulomatosis with polyangiitis
IVCY	Intravenous cyclophosphamide
MPA	Microscopic polyangiitis
mPSL	Methylprednisolone
PSL	Prednisolone
RD	Risk difference
SMD	Standardized mean difference
TMP-SMZ	Trimethoprim–sulfamethoxazole
