# Supplementary material for: The Role of Kidney Biopsy as a Tool for Personalized Treatment Decision-Making in Patients with Anti-Neutrophil Cytoplasmic Antibody (ANCA)-Associated Nephritis
Source: J Pers Med. 2026 Mar 7;16(3):153. doi: 10.3390/jpm16030153 (PMC13028148; doi:10.3390/jpm16030153)
Supplement: Supplementary file 1 [file jpm-16-00153-s001.zip › jpm-4126253-supplementary.pdf]

Supplementary Table 1. Details of the treatment

	Before overlap weighting					After overlap weighting				
	Kidney biopsy (-)		Kidney biopsy (+)		SMD	Kidney biopsy (-)		Kidney biopsy (+)		SMD
	N=36		N=38			N=10.3		N=10.3		
**Treatment pattern**										
PSL (maximum), mg/kg/day (SD)	0.70	(0.25)	0.69	(0.70)	0.12	0.66	(0.21)	0.68	(0.11)	0.13
Rapid PSL reduction, n (%)	17	(47.2)	15	(39.5)	0.16	3.7	(35.9)	4.9	(47.9)	0.25
Intensive immunosuppression, n (%)	19	(52.8)	32	(84.2)	0.72	5.7	(55.3)	8.7	(84.3)	0.66
PSL alone, n (%)	17	(47.2)	6	(15.8)	0.72	4.6	(44.7)	1.6	(15.7)	0.66
mPSL pulse, n (%)	17	(47.2)	31	(81.6)	0.77	5.3	(51.7)	7.9	(76.4)	0.53
Cyclophosphamide, n (%)	6	(16.7)	12	(31.6)	0.35	2.4	(23.0)	2.2	(21.4)	0.04
Rituximab, n (%)	1	(2.8)	5	(13.2)	0.39	0.0	(0.40)	1.5	(14.4)	0.56
Plasma exchange, n (%)	6	(16.7)	4	(10.5)	0.18	2.3	(22.4)	0.8	(8.1)	0.41
**Clinical events (within six months)**										
Death and ESKD, n (%)	14	(38.9)	11	(28.9)	0.21	3.8	(36.7)	3.8	(36.5)	0.01
Death, n (%)	9	(25.0)	4	(10.5)	0.39	1.9	(18.6)	1.5	(14.8)	0.10
ESKD, n (%)	8	(22.2)	7	(18.4)	0.10	3.0	(29.0)	2.2	(21.7)	0.17
Infectious complications, n (%)	13	(36.1)	7	(18.4)	0.41	4.8	(46.2)	2.1	(20.4)	0.57

The non-integer sample sizes after overlap weighting represent weighted pseudo-samples rather than actual patient counts. ESKD, end-stage kidney disease; mPSL, methylprednisolone; PSL, prednisolone; SD, standard deviation; SMD, standardized mean difference.

Supplementary Table 2. Association between kidney biopsy and clinical outcomes within six months and intensive immunosuppressive therapy before overlapped weighting based on the propensity score.

	Risk difference (%)	95%CI	*P*-value
Intensive immunosuppressive therapy	31.4%	0.106 - 0.509	0.003
Death or ESKD within six months	-9.9%	-0.313 - 0.119	0.37
Death within six months	-14.5%	-0.324 - 0.030	0.11
ESKD within six months	-3.8%	-0.227 - 0.149	0.69
Infectious complications within six months	-17.7%	-0.376 - 0.028	0.09

CI, confidence interval; ESKD, end-stage kidney disease.

Supplementary Table 3. Details of the treatment and outcome in each pathological classification based on Berden's classification in 27 patients with ANCA-associated nephritis who underwent kidney biopsy in Shinshu University Hospital.

Pathological classification	Focal (N=11)		Crescentic (N=3)		Mixed (N=10)		Sclerotic (N=3)		P-value
Treatment									
PSL (mg/kg)	0.74 ± 0.11		0.63 ± 0.14		0.75 ± 0.15		0.70 ± 0.14		0.55
mPSL (n, %)	9	81.8	3	100	10	100	2	66.7	0.30
Cyclophosphamide (n, %)	3	27.3	0	0.0	2	20.0	2	66.7	0.28
Rituximab (n, %)	1	9.1	0	0.0	3	33.3	1	33.3	0.40
Plasma exchange (n, %)	2	18.2	1	33.3	2	20.0	0	0.0	0.77
Outcomes									
Death (n, %)	0	0.0	0	0.0	1	10.0	0	0.0	0.62
Death or ESKD (n, %)	1	9.1	2	66.7	6	60.0	0	0.0	0.028
ESKD (n, %)	1	9.1	2	66.7	5	50.0	0	0.0	0.06
Infectious complications (n, %)	2	18.2	1	33.3	2	20.0	0	0.0	0.77

The pathological classification based on the Bernad classification in patients with ANCA-associated nephritis in patients who underwent kidney biopsy in Shinshu University Hospital.

Continuous variable among four groups was compared using the Kruskal-Wallis test. Categorical variables among the four groups were compared using the Chi-square test.

ESKD: end-stage kidney disease, mPSL: methylprednisolone, PSL: prednisolone.
